# Extracorporal Ovary Bioreactor System with Oxygen Supply and Drug Delivery Option

**DOI:** 10.3390/bioengineering12111158

**Published:** 2025-10-26

**Authors:** Karin Bundschu, Sabine Eberhart, Florian Maiss, Isabella Milic, Martin Hessling

**Affiliations:** 1 Department of Gynaecology and Obstetrics, University Hospital Ulm, Prittwitzstr. 43, 89075 Ulm, Germany; sabine.eberhart@uniklinik-ulm.de; 2 Institute of Medical Engineering and Mechatronics, Ulm University of Applied Sciences (Technische Hochschule Ulm), Albert-Einstein-Allee 55, 89081 Ulm, Germany; f.maiss@t-online.de (F.M.); isabella.milic@yahoo.de (I.M.)

**Keywords:** bovine ovary, perfusion bioreactor, long-time cultivation, follicle maturation, drug delivery system

## Abstract

Young women who have survived cancer may have lost their fertility due to cytotoxic treatments like chemotherapy or irradiation. So far, oocyte or ovarian tissue cryopreservation are well-known and well-used opportunities for fertility preservation prior cytotoxic therapies. However, these methods are not possible in certain cases, like those with a high risk of ovarian metastasis or prepubertal girls. Therefore, new medical and biotechnological options are also being sought to help this patient group to fulfill their desire to have their own biological children. The investigation described here focuses on the possibility of in vitro follicle maturing. To this point, a long-term temperature and pH-controlled bioreactor system is developed that can supply a whole ovary with oxygen and nutrients over several days and offers the possibility of hormone administration or the delivery of other drugs. This bioreactor was then tested with mature bovine ovaries. After appropriate cannulation, antithrombotic vascular perfusion, and antibiotic pretreatment, the ovaries were cultured for up to 9 days without any contamination or suffering major vital cell damage. The controlled application of oocyte stimulation hormones (human menopausal gonadotropin; hMG) also enabled successful in vitro follicle growth and maturation. From a technical point of view, there is still optimization potential for this bioreactor system, but in principle, it has been demonstrated that long-term ovary cultivation and in vitro maturation of follicles are possible, which opens up further potential for these and other applications.

## 1. Introduction

Ongoing advancements in oncological therapies have significantly improved long-term survival rates among young women diagnosed with malignant tumors. Breast cancer, as well as malignant gynecological and hematological neoplasms, are among the most common cancer diagnoses in women of reproductive age [1,2].

However, gonadotoxic treatments such as chemotherapy and radiotherapy frequently lead to irreversible damage to ovarian function, potentially resulting in permanent infertility [3]. As the average age of first-time mothers continues to rise in industrialized countries, many patients have not yet initiated or completed their family planning at the time of cancer diagnosis. Consequently, fertility preservation and post-treatment reproductive planning are gaining increasing relevance, aiming to ensure reproductive autonomy and provide cancer survivors with the possibility of biological parenthood [1]. Currently, fertility-preserving strategies during systemic cancer treatment are limited. Available options include temporary ovarian suppression with GnRH (Gonadotropin-Releasing Hormone) analogs during cytotoxic therapy and the cryopreservation of oocytes or ovarian tissue [4].

However, oocyte cryopreservation requires hormonal stimulation over several days, which may not be feasible in cases of urgent oncological intervention or hormone receptor-positive tumors. Furthermore, the reimplantation of cryopreserved ovarian tissue is generally contraindicated in patients with a high risk of ovarian metastasis, such as those with leukemia or certain ovarian malignancies [5,6,7]. At present, there are no validated options for fertility preservation in prepubertal girls undergoing cytotoxic therapy [8]. Given these limitations, in vitro follicle maturation and further innovative biotechnological approaches are highly desirable. In vitro follicle maturation has been the subject of research for a fairly long time. As early as 1938, Martinovich succeeded in maturing mouse and rat ovaries for up to 30 days in a kind of Petri dish [9]. However, corresponding successes in larger animals or humans are still rare. It is assumed that two-dimensional cell culture conditions are insufficient in these cases and that follicle maturation in larger animals requires a three-dimensional environmental structure [10,11,12], as found in the ovary itself.

The development of innovative in vitro culture methods for follicle maturation from cryopreserved ovarian tissue represents a major challenge in reproductive biology. Biotechnological strategies—such as 3D cultures, bioprinting, and scaffold systems—offer significant improvements over conventional 2D cultures. The physiological microarchitecture of the ovary also seems to be particularly important for preserving follicle morphology. Alkali et al. showed that the culture of vitrified bovine ovarian tissue on agarose gel inserts preserves the integrity of the follicles [13]. The composition of the extracellular matrix surrounding the follicles, as well as mechanical tension and signal factors, appear to be essential for functional follicle maturation [14,15]. Advances in functional follicle maturation have been demonstrated in the culture of preantral sheep follicles on 3D model scaffolds using polycaprolactone (PCL) [16]. Felder et al. used macroporous scaffolds with affinity-bound growth factors (e.g., BMP-4) to stimulate follicles [17]. Using Bio3D-printed microporous hydrogel scaffolds, a bioprosthetic ovary was created that restored both hormone function and fertility after implantation in sterilized mice [12]. Other bioprinting technologies have also been investigated in relation to ovarian tissue and oocyte maturation. Mastrorocco et al. developed an encapsulation method for cumulus oocyte complexes (COCs) (alginate-based COC microbeads) and demonstrated improved in vitro development of lamb oocytes using these artificial 3D systems [18,19]. Further studies showed that for vitrified cat oocytes, improved maturation results could be achieved in 3D culture models using granulosa cells or cumulus oocyte complexes (COCs) by mimicking the in vivo follicular environment [20,21,22]. Other factors also appear to play an important role in ovarian integrity and functionality. In vitrified cat ovarian tissue, it has been shown that melatonin supplementation can reduce oxidative stress and thus contribute to better tissue preservation [23]. Combelles et al. (2005) demonstrated the in vitro maturation of human oocytes in a co-culture with cumulus cells in a three-dimensional collagen gel system [24].

Additionally to physiological microarchitecture and ovarian niche factors, a continuous supply of nutrients and oxygen is necessary [25,26,27], as can be provided by a long-term extracorporeal in vitro bioreactor system using whole ovaries. Under controlled, sterile, and physiologically relevant conditions, ovarian tissue could be maintained viable and hormonally stimulated over several days, potentially allowing the in vitro maturation and retrieval of fertilization-competent metaphase II (MII) oocytes. A controlled and combined bioreactor perfusion and drug delivery system could provide a valuable foundation for this purpose. Initial successes have now been observed, among others, with sheep ovary strips by Maffei et al. in a setup in which a medium with or without hormones cycled through the ovary for up to 4 days and follicle growth was detected [28]. This system did not include an oxygen supply, nor did the perfusion bioreactor developed by Zanotelli et al. [29], in which complete bovine ovaries could be cultured for up to 2 days to perform toxicity studies. Tsiartis et al. and Hatekar et al. [30,31] applied a perfusion bioreactor for the cultivation of complete sheep ovaries with hormone administration and oxygenation of the medium for up to 8 days and achieved follicle maturation until the removal of mature oocytes.

Only some of the above-mentioned bioreactor systems for culturing the ovaries of large animals allowed for the oxygenation of the medium, even though oxygen concentration can have a major impact on follicle development. This is probably less relevant for shorter cultivation periods of a few hours, but necessary for periods lasting several days or longer. None of the systems seem to measure O_2_ concentration. Moreover, temperature and pH values also do not appear to be recorded.

Therefore, the investigation presented here aims to develop a perfusion bioreactor system for complete ovaries that allows for the oxygenation of the medium in order to reach or exceed the maximum cultivation time of 8 days achieved to date, as follicle maturation takes longer in many large animals, including humans. The actual O_2_ concentration in the medium was monitored continuously, as were pH and temperature. Furthermore, the automatic delivery of drugs, in this case hormones, to stimulate follicle growth, was integrated, and it was possible to change the medium automatically or manually as needed to enable longer cultivation times. This was tested on the cultivation of bovine ovaries and the recording of follicle growth after hormone application.

## 2. Materials and Methods

### 2.1. Ovary Bioreactor

**Figure 1:**
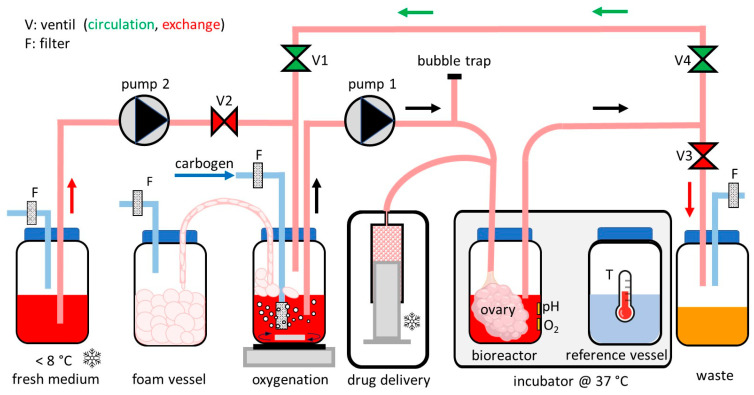
Schematic setup of the ovary bioreactor system. Two peristaltic pumps (pump 1 and pump 2) and four pinch valves (V1–V4) allow for different media flows. The central circuit contains the ovary in the bioreactor in the 37 °C incubator, the oxygenation vessel, and pump 1. Sterile filters (F) are installed at various points. Hormones or other liquid substances can be injected into this central circuit via the temperature-controlled drug delivery unit. The two vessels on the left contain fresh medium and collect foam that can form during the oxygenation process. The container on the far right is a waste vessel for exhausted medium. The vessel on the right of the incubator is intended as reference for sterile temperature measurement to ensure sterility in the main bioreactor.

The aim is to create a perfusion bioreactor for the long-term cultivation of a whole bovine ovary by supplying the tissue with appropriate nutrients and oxygen, as illustrated schematically in Figure 1. The bovine ovary is located in a bioreactor and is connected to a medium circuit via the main ovarian artery, as described in Section 2.2. There is a total of approximately 2 L of medium in the entire system, distributed among various vessels and tubes. The tubes are Masterflex C-Flex silicone tubes with an inner diameter of 1.7 mm (Cole-Parmer Instrument, Vernon Hills, IL, USA).

The central circuit of the fluidic system comprises the ovary in the bioreactor, the oxygenation unit, and a PD 5201 peristaltic pump (Heidolph, Schwabach, Germany), which transports the medium at a set speed of 1.5 mL/min. The actual bioreactor is a 500 mL borosilicate glass bottle, which is kept at a temperature of 37 °C in a homemade incubator with transparent plastic walls. Inside the bottle are autoclavable glued-in sensor spots for measuring pH and O_2_ concentration, which can be read optically from the outside using the pH-1 mini and Fibox 3 measuring devices (both from PreSens, Regensburg, Germany). In order not to compromise sterility in the bioreactor, the actual temperature measurement is not carried out in the bioreactor itself but with the aid of a PreSens thermometer located in a reference bottle of similar volume in the same incubator.

Another element of the central circuit is the oxygenation unit. This is provided by an additional glass bottle located outside the incubator. A mass flow controller type F-201CV-020-AAD-33V (Bronkhorst High-Tech, Ruurlo, The Netherlands) is used to add 16 mL/min of carbon dioxide gas with 95% O_2_ and 5% CO_2_ (Linde, Pullach, Germany) to the medium in the oxygenation container. A stainless-steel diffuser with a pore size of 0.5 µm (Mirthful Minstrels, China) generates many small gas bubbles, which, together with the lower temperature, facilitates gas transfer into the medium. A stirring fish (VWR, Darmstadt, Germany) in the medium and an MR 1000 magnetic stirrer (Heidolph, Schwabach, Germany) under the vessel ensure a more even distribution of the gas bubbles in the medium and thus of the O_2_ concentration. Another connected vessel collects the foam produced during oxygenation to avoid microbial contamination, and a bubble trap between the oxygenation unit and the ovary bioreactor vessel prevents bubbles from circulating in the medium.

With the assistance of four pinch valves of type 100P2NC12-03BQ (Bio-Chem Fluidics, Boonton, NJ, USA) and an additional peristaltic pump of type PD 5001 (Heidolph, Schwabach, Germany), other medium flows can also be achieved. For example, valve 2 can be used to feed cooled, fresh medium from an ice-cooled storage tank into the central circuit, and valve 3 can be used to pump depleted medium from the circuit into a waste container.

In addition, it is possible to automatically deliver hormones or other drugs into the central medium circuit using a syringe pump LA110 (Landgraf Laborsysteme, Langenhagen, Germany). This pump and a 20 mL syringe (Omnifix Luer Solo, B. Braun, Melsungen, Germany) are located outside the incubator in a mini refrigerator model K-MKS-2076 (Kesser, Altenberge, Germany), which is cooled to approximately 5 °C.

All functions are implemented using the LabVIEW programming language (version 2024 Q3, National Instruments, Austin, TX, USA) on an older PC model Esprimo p920 e85+ (Fujitsu, Munich, Germany). The LabVIEW user interface allows various parameters to be set and functions such as media changes to be triggered manually or automatically. Most of the inputs and outputs are handled by DAQ Modules 6001 and 6008 USB I/O cards (National Instruments, Austin, TX, USA). Parallel to the self-written LabVIEW software, Presens programs run to record pH and O_2_ concentrations in the ovary bioreactor.

All components of the bioreactor system are cleaned and autoclaved before each cultivation cycle in accordance with WHO (World Health Organization) and RKI (Robert Koch Institut) recommendations [32,33] and assembled under a sterile workbench. To protect the environment and conserve general resources, plastic and glass materials were reused wherever possible and autoclaved before each reuse.

### 2.2. Ovary Preparation, Cultivation, and Analysis

Bovine ovaries are kindly provided by Maucher slaughterhouse (Illertissen, Germany) and dissected together with the surrounding tissue right after the animal’s death. Immediate and rapid preparation is essential to prevent contamination and shorten the cold ischemia time. Cannulation and initial flushing should be performed no later than 1.5 h after the animal’s death in order to prevent (micro-)thrombosis and subsequent ischemia in these tissue areas. The ovaries are considered as waste material during slaughter, so no animal welfare application is required for these experiments.

**Figure 2:**
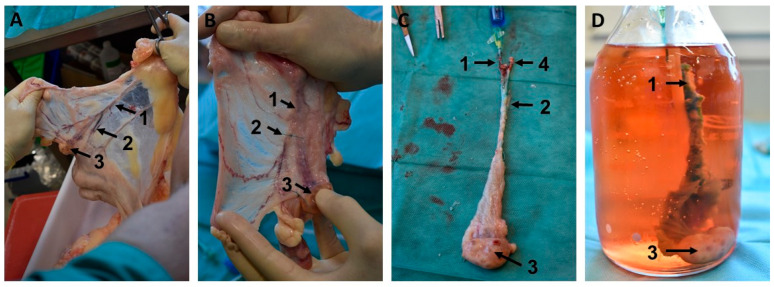
Bovine ovary preparation for bioreactor cultivation: (**A**) anatomical identification of the arteria ovarica (1), the Ramus uterinus (2) and the ovary (3) within the mesovar. (**B**) Ligation of the Ramus uterinus (2). (**C**) Cannulation of the A. ovarica (1) and perfusion test of the ovary (3) with blue solution flow out of the V. ovarica, after removal of mesovar tissue. Bovine ovary in the bioreactor (**D**). (1: A. ovarica; 2: Ramus uterinus; 3: ovary; 4: V. ovarica).

The following treatment is based on the procedure described by Tsiartis et al. [30]. First, the ovaries are separated from the surrounding connective tissue complex, disinfected in a 0.001% chlorhexidine solution, and then transferred and washed in a container with sterile PBS (phosphate-buffered saline). The different steps of further preparation are depicted in Figure 2. They are performed on a sterile working platform with sterile preparation equipment to avoid contamination. For vascular cannulation, the ovarian artery is dissected with magnifying glasses proximal to the ramus ovaricus of the A. ovarica by using a flexible vascular cannula (Vasofix Safety, B. Braun, Melsungen, Germany) that is securely fixed by applying surgical suture material (Mersilene; Ethicon, Johnson & Johnson Medical, Norderstedt, Germany). In order to perfuse the corkscrew-like bovine A. ovarica, the surrounding connective tissue is carefully removed with fine tweezers. This allows the artery to be stretched along its course and cannulated with the flexible plastic cannula.

The vascular system is first flushed with PBS containing heparin (50 IU/mL, ratiopharm GmbH, Ulm, Germany) and the following antimicrobial additives: Lidocaine (0.04 mg/mL, Deltamedica, Reutlingen, Germany), “PipTaz” (piperacillin 10 µg/L, tazobactam 1.25 µg/L: Fresenius Kabi, Bad Homburg, Germany), and “Anti-Anti” (penicillin 10 IU/mL, streptomycin 100 µg/L, amphotericin B 250 ng/L, Gibco/ThermoFisher, Darmstadt, Germany) to prevent vascular thrombosis and microbial contamination.

A blue solution is injected to identify proper vascular and ovarian organ perfusion and for the detection of potential leakages. For this purpose, an ampoule containing 0.8% indigo carmine (Provendo, Provepharm, Heathrow, UK) is diluted with a 0.9% sodium chloride solution (NaCl, B. Braun, Melsungen, Germany). As illustrated in Figure 2, the venous outlet remains open, while all other arterial vascular branches (R. uterinus and R. ovaricus of the A. ovarica) are carefully ligated by using surgical suture material (Mersilene; Ethicon, Johnson & Johnson Medical, Norderstedt, Germany).

After successful cannulation and careful ligation of possible side branches and leaks, the ovary is transferred sterile to the bioreactor vessel, filled with cold perfusion medium, and sealed airtight. Transport to the laboratory is carried out under refrigerated conditions (transportation time approximately 30–60 min). There, the bioreactor container is connected to the perfusion system and the experiment starts. Immediate preparation after circulatory arrest in the animal allows the ischemia time to be kept below three hours.

After starting the bioreactor in the laboratory, the ovary is supplied with medium as described in Section 2.1. The selected medium is DMEM (Dulbecco’s Modified Eagle Medium) F12 with stable glutamine and HEPES (VWR, Darmstadt, Germany) for pH stabilization, with an additional 100 mL fetal bovine serum (FBS, Gibco/Thermo Fisher Scientific, Hampton, VA, USA) per liter of DMEM. The medium also contains the antimicrobial additives mentioned above: Lidocaine (0.04 mg/mL, Deltamedica, Reutlingen, Germany), “PipTaz” (piperacillin 10 µg/L, tazobactam 1.25 µg/L: Fresenius Kabi, Bad Homburg, Germany), and “Anti-Anti” (penicillin 10 IU/mL, streptomycin 100 µg/L, amphotericin B 250 ng/L, Gibco/ThermoFisher, Darmstadt, Germany). The necessary use of antibiotics was adjusted during the experimental course. Since the initial application of penicillin/streptomycin alone was insufficient and contamination occurred during experiments, which lasted several days, broad-spectrum antibiotics were used, as described in Tsiartis et al. [30].

Menogon HP 600 IU (Ferring Pharmaceuticals, Kiel, Germany) is injected to stimulate hormone production. Menogon HP contains highly purified human menopausal gonadotropin (hMG), a mixture of follicle-stimulating hormone (FSH), and luteinizing hormone (LH). It is used to treat infertility in women with ovulation problems or for ovarian stimulation in IVF therapies (in vitro fertilization). The hormone preparation is diluted to 50 IU per ml in the DMEM F-12 perfusion medium, resulting in a total volume of 12 mL, which is loaded into the syringe pump. At two 4 h intervals, 125 µL/h of the diluted hormone solution is delivered into the central circulation. The total daily dose of hMG is therefore calculated as 50 IU.

Cultivation continues until clear growth of follicles can be observed on the ovarian surface in the bioreactor. The ovary is then removed and analyzed as follows: histology (HE staining) and immunohistochemical staining is performed to assess cell viability and tissue condition after perfusion. The tissue samples are first fixed in formalin and then embedded in paraffin. The paraffin sections are deparaffinized for staining, first in Neo-Clear (Sigma Aldrich, Darmstadt, Germany) and then rehydrated using a descending alcohol series. This is followed by washing in deionized water and buffering in Tris solution (pH 7.6). For antigen demasking, the sections are heated in citrate buffer (pH 6.0) in a pressure cooker for four minutes. After cooling to room temperature, they are transferred to a Tris-buffered saline (TBS) solution to equalize the buffer ratio. To reduce nonspecific background staining, endogenous peroxidase activity is inactivated by treatment with a hydrogen peroxide–methanol solution. The sections are then outlined with a fat pen (PAP pen, Merck, Darmstadt, Germany). A goat serum-based block buffer is applied to block non-specific binding sites. Incubation is carried out with specific primary antibodies that are specifically directed against the respective target antigens.

Antibodies against cleaved caspase-3 (anti-rabbit; 9579, Cell Signaling Technology, Danvers, MA, USA) and Ki67 (anti-mouse; 9449, Cell Signaling Technology, Danvers, MA, USA) are used. Ki67 serves as a marker for cell proliferation, while cleaved caspase-3 indicates apoptotic processes. After a washing step with TBS, the corresponding secondary antibodies are applied.

The antigen–antibody complexes are visualized using streptavidin-coupled horseradish peroxidase (HRP) and diaminobenzidine (DAB), both of ThermoFisher, Darmstadt, Germany. Finally, the sections are rinsed under running water and stored in the dark until microscopic evaluation.

## 3. Results

### 3.1. Ovary Bioreactor

**Figure 3:**
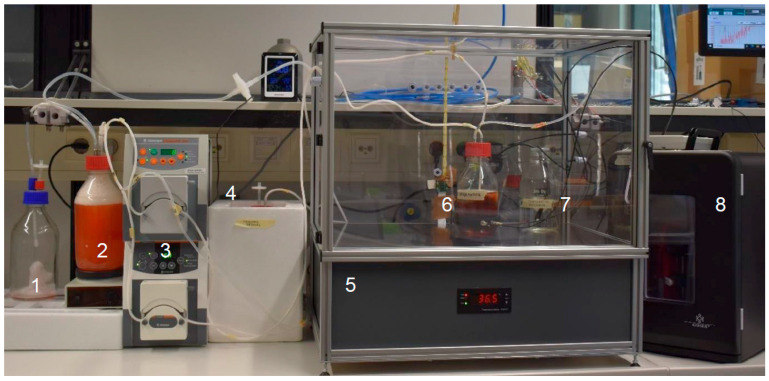
Setup of the bioreactor system: foam container (1), oxygenation vessel (2), feed pumps (3), a cooled area with the reservoir for fresh medium (4), the incubator (5), the perfusion bioreactor with ovary (6), the reference vessel (7), and the mini refrigerator with an integrated drug delivery system (8).

The actual bioreactor system with bovine ovary is shown in Figure 3. A total of more than 10 cultivation experiments were carried out with this system. The first experiments were performed with a different, less effective antibiotic, which led to frequent contamination and premature termination of the cultivation period. Furthermore, delayed cannulation and perfusion of the ovaries resulted in vessel thrombosis and thereby inadequate ovarian perfusion when the interval between the animal’s death and the actual dissection was too long. Further complications arose due to an initially undetected ventilation problem in the laboratory being used, which led to very high room temperatures of over 30 °C and made it difficult to regulate the temperature in the transparent incubator.

**Figure 4:**
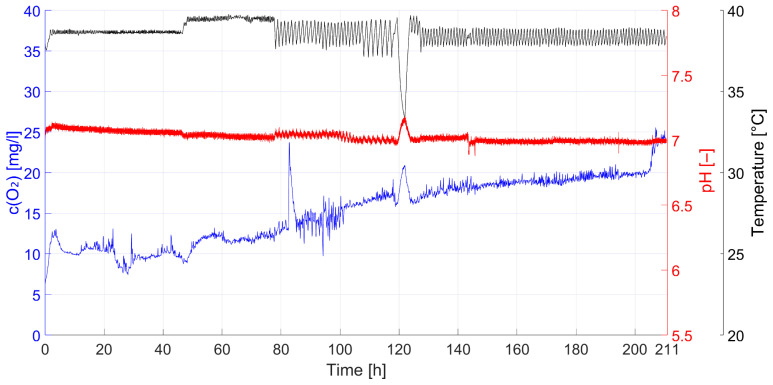
Bio-parameter measurements: temperature (black), pH (red), and O_2_ concentration (blue) over a cultivation period of 211 h.

The longest cultivation period lasted 211 h, or just under 9 days, and was terminated when mature follicles were visible macroscopically by eye. The values recorded for temperature, pH, and O_2_ concentration during this period are presented in Figure 4. The pH value remained relatively constant at seven. The average temperature was approximately 37 °C, but exhibited slight fluctuations and, after approximately 120 h, a brief temporary drop to approximately 27 °C for unknown reasons. The O_2_ concentration also exhibited some fluctuations, as well as a general increase over the course of the cultivation period. The cause of this might be the oxygenator. On the one hand, the stirrer driven by the magnetic plate did not always rotate evenly and even stopped in exceptional cases. On the other hand, the suction effect of the stirrer also influenced the position of the sparger, which was not fixed but hung on a flexible hose.

### 3.2. Ovary Cultivation and Analysis

**Figure 5:**
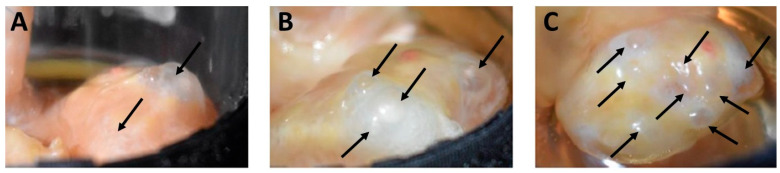
Macroscopic examination of ovaries in the bioreactor following treatment with follicle-stimulating hormones. Ovarian surface with developing follicles (arrows) during cultivation and hormone administration after 6 (**A**), 7 (**B**), and 8 (**C**) days, or after 250 (**A**), 300 (**B**), and 350 (**C**) IU hMG.

During bioreactor cultivation, follicle growth was monitored, detectable by visual follicle appearance on the surface of the ovary. The temporal course of observed follicle development and growth after hormone administration is given in Figure 5. In bioreactor cultivations without any administration of hormones, there was no comparable follicle development detectable. The ovary was then removed, re-tested for appropriate perfusion with blue solution, oocytes were harvested from the follicles, and ovarian tissue was examined histologically and immunohistochemically.

**Figure 6:**
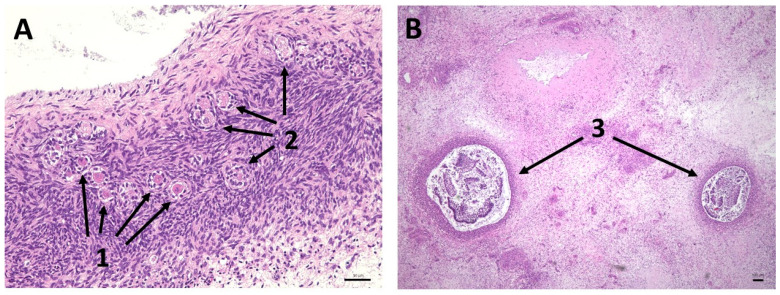
Histological (HE) staining of the harvested ovary after 211 h of bioreactor cultivation. (**A**,**B**) Physiologically intact bovine ovarian tissue, displaying various follicle stages: primordial follicles (1), primary follicles (2), and antral follicles (3) after hMG stimulation. Scale bars (**A**) 50 µm, (**B**) 100 µm.

**Figure 7:**
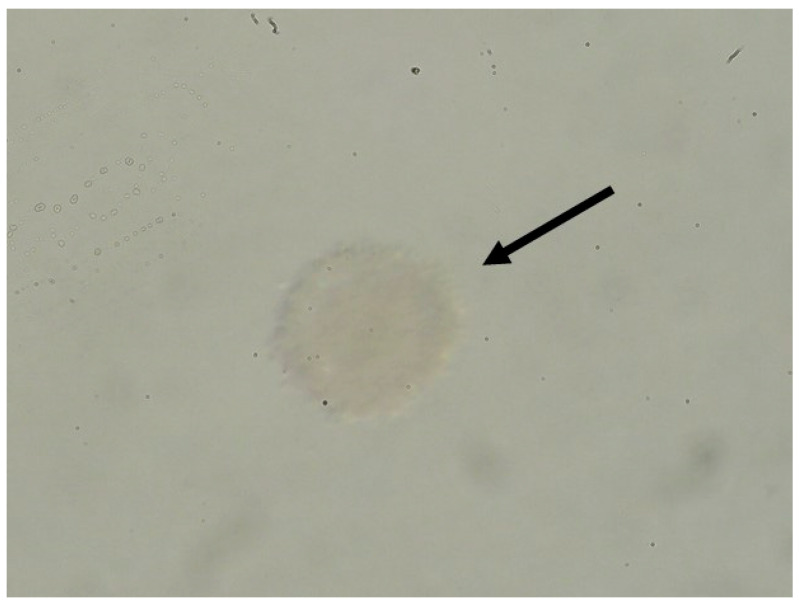
Microscopic image of an aspirated oocyte (arrow), harvested after almost 9 days of bioreactor cultivation and hormonal stimulation with 350 IU hMG.

The histological microscope images (HE staining) in Figure 6 reveal different preserved follicular structures in the ovarian tissue. Primordial, primary, and antral follicles are recognizable here. The histologically examined tissue quality of the ovaries after cultivation in the bioreactor was comparable to that of bovine ovaries preserved immediately after slaughter. Figure 7 is a microscopic image of a punctured follicle. The isolated oocyte demonstrates successful oocyte aspiration.

**Figure 8:**
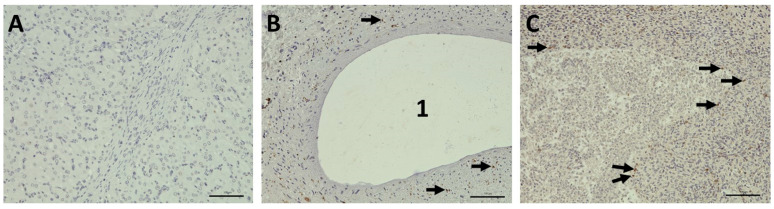
Apoptosis shown by immunohistochemical staining of cleaved caspase-3 in bovine tissue. (**A**) Vital ovarian tissue, immediately after harvesting in the slaughterhouse; (**B**,**C**) rare apoptosis after 211 h cultivation in the bioreactor. Arrows pointing to apoptotic/cleaved caspase-3-positive cells. (1) Antral follicle. Scale bars 100 µm.

Figure 8 depicts apoptosis (cleave caspase-3 staining) for an ovary immediately harvested after removal at the slaughterhouse (Figure 8A) and an ovary after 211 h in the bioreactor (Figure 8B,C). In fact, more apoptotic cells can be found in the ovary following bioreactor cultivation, but the overall apoptotic cell proportion even in this tissue is rather low.

**Figure 9:**
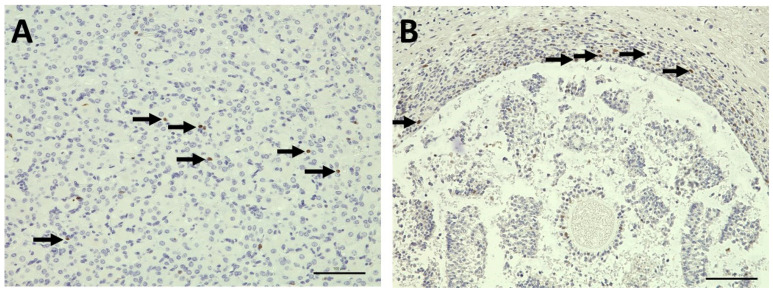
Cell proliferation shown by immunohistochemical staining of Ki67 in bovine tissue. (**A**) Immediately after harvesting in the slaughterhouse; (**B**) antral follicle after 211 h cultivation in the bioreactor. Arrows pointing to Ki67-positive cells. Scale bars 100 µm.

A similar comparison between the ovary after removal (Figure 9A) and after 211 h of cultivation in the bioreactor (Figure 9B) is provided by Ki67 staining in Figure 9. The Ki67-positive cell nuclei indicate proliferative areas. These are fewer in the bioreactor ovary, but still present even after 211 h.

## 4. Discussion and Conclusions

Previous studies have already explored the use of bioreactors to culture whole ovaries ex vivo, aiming to preserve ovarian function, support oocyte maturation, or assess toxic effects of drugs in a controlled environment [28,29,30,31]. These systems offer a promising platform for fertility preservation and reproductive toxicology. Whole-organ perfusion models preserve the 3D architecture and vascular supply of the ovary, allowing more physiological conditions than isolated follicle cultures. Zanotelli et al. described a perfused bovine ovary model and maintained tissue viability for up to 48 h [29]. They also showed the model’s suitability for drug testing, using doxorubicin to induce apoptosis. Longer-term cultures have also been achieved in sheep. In a recent study, Hatekar et al. [31] perfused whole ewe ovaries for 4–8 days. Depending on the gonadotropin protocol, they retrieved both immature and mature oocytes (including MII), along with functional hormone production and relatively stable follicular morphology. However, extended culture increases the risk of apoptosis and tissue damage, especially with continuous stimulation. One of the most critical steps is perfusion timing and delays before cannulation, which significantly reduce perfusion efficiency and increase tissue damage, underlining the importance of rapid post-mortem handling. Immediate dissection and arterial cannulation directly after the animal’s death at the slaughterhouse is essential to prevent vascular thrombosis and to minimize cold ischemia time. Moreover, sterile working conditions and the use of appropriate antibiotics are essential to prevent microbial contamination within the whole preparation and bioreactor process.

In this study, we demonstrate the functionality of our established bioreactor system, which supports the cultivation of bovine ovaries for up to nine days. Long-term tissue viability is enabled by continuously controlled oxygenation and the ability to exchange the culture medium. While parameters such as pH, temperature, and oxygen concentration are not yet as stable as intended, initial areas for technical improvement have been identified. For instance, optimization of the integrated stirrer and sparger holder is necessary to enhance oxygen delivery. This could enable precise control of oxygen concentrations via the existing carbogen gas mass flow controller. Notably, to our knowledge, this study is the first to include real-time monitoring of oxygen levels in an ovarian bioreactor system. In previous studies, including those that incorporated oxygenation features, oxygen concentration was not directly measured, leaving the stability of O_2_ levels during culture uncertain. Moreover, our temperature-controlled drug delivery system worked satisfactorily.

The programmed addition of hormones proved effective: follicular development was observed following the administration of hMG, whereas no such development occurred in its absence.

To our knowledge, this is the first time that such measurements have been conducted in an ovarian bioreactor, and the same applies to the possibility of medium exchange and drug delivery. With our setup, we also have the long-term possibility of setting desired the O_2_ concentrations, e.g., by the carbogen mass flow controller, or even culturing multiple ovaries simultaneously.

In current clinical practice for fertility preservation in humans, either oocytes obtained after in vivo stimulation therapy or surgically obtained fragments of ovarian tissue are cryopreserved. Possible approaches, including the cryopreservation of whole ovaries, have been investigated in sheep [34,35] and bovine animal models [36], so that in the future it may also be possible to cryopreserve entire human ovaries in such a bioreactor system for follicle stimulation. Initial approaches to the cryopreservation of whole human ovaries have already been published by Martinez-Madrid et al. [37,38]. However, whole human ovaries cannot yet be reliably cryopreserved because the diffusion of cryoprotectants and thus the preservation of vital tissue is insufficient.

Under stable, reproducible bioreactor conditions, human ovaries obtained surgically could also be used directly in a bioreactor system for cultivation and multiple stimulation in order to obtain and cryopreserve mature oocytes for fertility preservation. This would be particularly attractive if there is not enough time for in vivo stimulation therapy due to high oncological therapy pressure. Furthermore, transplantation experiments involving human ovarian tissue fragments on a bovine ovary as described here for stimulation in a bioreactor would also be conceivable. Such an ovarian bioreactor system would also be particularly interesting for testing the toxicity of pharmaceutical agents on oocytes or for testing alternative substances for stimulating oocytes. This could also lead to a sustainable and relevant reduction in animal experiments, otherwise necessary for these purposes.

As mentioned above, more than 10 cultivations in the bioreactor system were carried out, but more than half of them had to be terminated prematurely due to initial microbial contamination. Due to limited personnel capacity, no more than four rounds of improved cannulation and antibiotic treatment could be performed over a longer period of time. Therefore, no reliable quantitative statistical analyses are currently available, which should be the aim of future studies.

The following challenges remain, cannulation is technically demanding, flow rates must be optimized to avoid edema or necrosis, and the follicle population within the ovary is heterogeneous. Moreover, maturation rates of oocytes are still limited in many systems, and reproducibility is a key barrier to translation. Despite these limitations, the potential applications are broad. These include not only fertility preservation in cancer patients, but also toxicological testing, drug screening, and the potential development of fully ex vivo oocyte maturation protocols. Future biotechnological work should focus on optimizing O_2_ and temperature control and controlled filling level in the bioreactor container. Furthermore, improved imaging of follicular development through enhanced camera systems may enable more accurate monitoring—potentially allowing for AI-assisted observations and evaluations in future applications.

## Data Availability

The original contributions presented in the study are included in the article, further inquiries can be directed to the corresponding author.
